# Integrated Youth Mental Health Services – niederschwellige, integrierte Angebote für junge Menschen in psychischen Krisen: Internationale Erfahrungen und die aktuelle Situation in Deutschland

**DOI:** 10.1007/s00115-025-01895-7

**Published:** 2025-09-04

**Authors:** Andreas Bechdolf, Christoph U. Correll, Tobias Hellenschmidt, Laura Holzner, Laura von Hardenberg, Dorothea Jäckel, Joseph Kambeitz, Nikolaus Koutsouleris, Norma Kusserow, Karolina Leopold, Eva Meisenzahl, Andrea Pfennig, Andreas Reif, Ullrich Reininghaus, Mario Schellong, Olga Shmuilovich, Peter J. Uhlhaas, Olga Maria Domanska

**Affiliations:** 1 Kliniken für Psychiatrie, Psychotherapie und Psychosomatik mit FRITZ am Urban & soulspace, Vivantes Klinikum Am Urban und Vivantes Klinikum im Friedrichshain, Dieffenbachstraße 1, 10967 Berlin, Deutschland; 2 Klinik für Psychiatrie und Psychotherapie, CCM, Charité – Universitätsmedizin Berlin, corporate member of Freie Universität Berlin and Humboldt-Universität zu Berlin, Berlin, Deutschland; 3 Deutsches Zentrum für Psychische Gesundheit (DZPG), Standort Berlin-Potsdam, Berlin, Deutschland; 4 Klinik für Psychiatrie, Psychosomatik und Psychotherapie des Kindes- und Jugendalters, CVK, Charité – Universitätsmedizin Berlin, corporate member of Freie Universität Berlin and Humboldt-Universität zu Berlin, Berlin, Deutschland; 5 Klinik für Kinder- und Jugendpsychiatrie, Psychotherapie und Psychosomatik, Vivantes Klinikum Friedrichshain und Neukölln, Berlin, Deutschland; 6 Klinik und Poliklinik für Psychiatrie und Psychotherapie, Uniklinik Köln, Köln, Deutschland; 7 Klinik und Poliklinik für Psychiatrie und Psychotherapie, Ludwig-Maximilians-Universität München, München, Deutschland; 8 Berliner Senatsverwaltung für Wissenschaft, Gesundheit, Pflege und Gleichstellung, Berlin, Deutschland; 9 Klinik und Poliklinik für Psychiatrie und Psychotherapie, Universitätsklinikum Carl Gustav Carus und Medizinische Fakultät der Technischen Universität Dresden, Dresden, Deutschland; 10 Klinik und Poliklinik für Psychiatrie und Psychotherapie, Heinrich-Heine-Universität, LVR Düsseldorf, Düsseldorf, Deutschland; 11 Klinik für Psychiatrie, Psychosomatik und Psychotherapie, Universitätsmedizin Frankfurt, Frankfurt am Main, Deutschland; 12 Abteilung Public Mental Health, Zentralinstitut für Seelische Gesundheit, Mannheim, Deutschland; 13 ajb GmbH – Gemeinnützige Gesellschaft für Jugendberatung und psychosoziale Rehabilitation, Berlin, Deutschland

**Keywords:** Niederschwellige Gesundheitsversorgung, Psychische Krisen/Früherkennung, Psychische Krisen/Frühintervention, Soulspace, Headspace, Health care, low-threshold, Mental health crisis/early detection, Mental health crisis/early intervention, Soulspace, Headspace

## Abstract

**Hintergrund:**

Junge Menschen in psychischen Krisen haben keinen einfachen Zugang zur klinischen Versorgung und zeigen die geringste Adhärenz zu den konventionellen klinischen Angeboten. Um diese Barrieren abzubauen und eine jugendgerechte Früherkennung, Frühintervention und psychosoziale Versorgung zu ermöglichen, werden niederschwellige und integrierte Angebote (Integrated Youth Mental Health Services [IYMHS]) international empfohlen.

**Ziel der Arbeit:**

Ziel der Arbeit ist es, einen Überblick über die niederschwelligen IYMHS-Angebote weltweit und in Deutschland zu geben. Zudem sollen die internationalen Erfahrungen in der Inanspruchnahme berichtet und die Evidenz zur Wirksamkeit von IYMHS zusammengefasst werden.

**Methoden:**

Die narrative Übersichtsarbeit basiert auf Recherchen in PubMed inklusive der Nutzung publizierter Reviews.

**Ergebnisse:**

Internationale Entwicklungen zeigen, dass sich IYMHS als innovative Versorgungsmodelle für junge Menschen in psychischen Krisen etabliert haben. Australien gilt mit seinem landesweiten Netzwerk von headspace-Zentren als Vorreiter. In Deutschland gibt es mit soulspace ein erstes Modellprojekt, ein weiteres – ancora – befindet sich im Aufbau. Bisherige Evaluationen zeigen, dass viele junge Menschen, die sonst keine Hilfe in Anspruch genommen hätten, diese integrierten Angebote nutzen und überwiegend symptomatische und funktionelle Verbesserung erfahren.

**Schlussfolgerung:**

Die bisherigen internationalen Erfahrungen und Pilotprojekte in Deutschland zeigen das Potenzial der IYMHS, jungen Menschen mit ersten psychischen Symptomen frühzeitig und niederschwellig Unterstützung anzubieten. Für Deutschland wird der Ausbau einer integrierten, sektorenübergreifenden Versorgungsstruktur empfohlen, realisiert durch die Bündelung von Mitteln der gesetzlichen Krankenversicherung und von öffentlichen Fördermitteln.

## Abstract (en)

**Background:**

Young people in mental health crises have severely limited access to clinical care and show the lowest adherence to conventional clinical services. Low-threshold and integrated services (integrated youth mental health services, IYMHS) are recommended to overcome these barriers and provide early detection, early intervention and psychosocial care appropriate for young people.

**Objective:**

The aim is to provide an overview of low-threshold IYMHS globally and in Germany and to report on international experiences with these services and evidence of their effectiveness.

**Methods:**

The narrative review is based on research conducted in PubMed, including the use of published reviews.

**Results:**

International developments show that IYMHS have become established as innovative models of care for young people in mental health crises. Australia is seen as a pioneer with its nationwide network of headspace centers. In Germany, *soulspace* is the first model project and another, *ancora*, is in the pipeline. Evaluations to date show that many young people who would not otherwise have sought help use these integrated services and predominantly experience symptomatic and functional improvements.

**Conclusion:**

International experiences to date and pilot projects in Germany have demonstrated the potential of IYMHS to provide low-threshold support at an early disease stage to young people with first symptoms of mental health. For Germany, expanding an integrated, cross-sectoral care structure is recommended through pooling resources from statutory health insurances and public funding.

Psychische Störungen stellen für die Betroffenen und die Gesellschaft eine große Herausforderung dar und gehören unter allen Erkrankungen zu denen mit der höchsten Krankheitslast [14]. Obwohl die Therapieeffekte bei voll ausgebildeten Störungsbildern (etwa bei schweren oder chronischen psychischen Erkrankungen) im Erwachsenenalter nur moderat sind, steht bisher die Behandlung dieser Krankheitsstadien im Vordergrund der Versorgung, während Frühintervention und Prävention vernachlässigt werden [13, 21]. Übereinstimmend kann anhand epidemiologischer Daten gezeigt werden, dass 62,5 % der psychischen Störungen vor dem fünfundzwanzigsten Lebensjahr beginnen [32]. Hinzu kommen Hinweise, dass die Inzidenzen psychischer Erkrankungen bei Jugendlichen und jungen Erwachsenen in den letzten zwei Jahrzehnten weltweit angestiegen sind, gegebenenfalls zusätzlich verstärkt durch die Coronavirus-disease-2019(COVID-19)-Pandemie [25]. Weitere Befunde sprechen für eine „sensitive Phase“ der Gehirn- und psychosozialen Entwicklung im Jugend- und jungen Erwachsenenalter, die für die langfristige psychosoziale Entwicklung der jungen Menschen besonders bedeutsam ist. Darüber hinaus zeigen zahlreiche Studien, dass eine Frühintervention bei psychischen Störungen im Jugend- und jungen Erwachsenenalter besonders effektiv ist [13]. Trotzdem haben Jugendliche und junge Erwachsene den schlechtesten Zugang zur klinischen Versorgung und zeigen die geringste Adhärenz zu den konventionellen klinischen Angeboten [27].

Vor diesem Hintergrund formulieren viele namenhafte Expertinnen und Experten – unter anderem Kommissionen des Weltwirtschaftsforums (World Economic Forum [WEF]) und der Fachzeitschrift *Lancet* [18, 25] – die Notwendigkeit eines Paradigmenwechsels in Psychiatrie und Psychotherapie hin zu einem Fokus auf „youth mental health“. Sie fordern eine Anpassung der klinischen Versorgungsangebote an den Bedarf junger Menschen [18, 25, 35]. Die Kommission aus Expertinnen und Experten des WEF, der auch junge Betroffene angehörten, erarbeitete die Prinzipien (Tab. 1) für jugendfreundliche, niederschwellige und integrierte Versorgungsangebote für junge Menschen in psychischen Krisen (Integrated Youth Mental Health Services [IYMHS]).

**Tab. 1:** Empfehlungen zu Strukturmerkmalen der Integrated Youth Mental Health Services (IYMHS). (Adaptiert nach World Economic Forum [18])

Öffentlichkeits- und Awareness-Arbeit für seelische Gesundheit
Beteiligung junger Menschen bei der Angebotsgestaltung und dem klinischen Angebot (Peer-Mitarbeitende)
Niederschwelliger, kostenloser, überweisungsfreier Zugang zu nichtstigmatisierenden Anlaufstellen in der Gemeinde (möglichst mit aufsuchender Komponente)
Optimistischer Ansatz mit Fokus auf Frühintervention
Integration von Kinder‑, Jugend- und Erwachsenenpsychiatrie und -psychotherapie (transitionspsychiatrischer „one-stop shop“)
Integration von psychiatrisch-psychotherapeutischen, Schul‑/Ausbildungs- und Arbeitsangeboten („supported employment and education“ [SEE]), Suchttherapie und (somatischen) Gesundheitsangeboten (interventioneller „one-stop shop“)
Enge Zusammenarbeit mit Schulen und Ausbildungsstätten
Einbezug von Angehörigen (Angehörigenangebote, Angehörigen-Peers)
Digitale Interventionsangebote
Inklusiv, kultursensibel und an Entwicklungsstadien angepasst

Auf Basis dieser Strukturmerkmale wird davon ausgegangen, dass die IYMHS für Betroffene attraktiver, akzeptabler und effektiver sind und somit langfristig zu besseren Krankheitsverläufen führen als konventionelle klinische Versorgungsstrukturen, die auf die Behandlung voll ausgebildeter Störungen im Erwachsenenalter ausgerichtet sind.

Ziel der vorliegenden Arbeit ist es, einen Überblick über bestehende IYMHS weltweit und in Deutschland zu geben. Darüber hinaus soll über internationale Erfahrungen mit der Inanspruchnahme und über die Evidenz zur Wirksamkeit dieser Angebote berichtet werden.

## Methode

Diese narrative Übersichtsarbeit wurde auf der Grundlage der Reviews von Hetrick et al. [15], Settipani et al. [30] und der Lancet Psychiatry Commission on Youth Mental Health [25] erstellt. Eine zusätzliche Literaturrecherche in PubMed wurde für Publikationen nach 2023 durchgeführt, mit Suchbegriffen in Anlehnung an die beiden Reviews: *mental youth services, youth mental health services, community mental health services, integrated mental healthcare, integrated youth mental health services*, youth, teenager*, young adult*, emerging adults, walk-in centre, one-stop shop.

## Ergebnisse

### Angebote von Integrated Youth Mental Health Services weltweit

Einen Überblick über die Angebote von IYMHS weltweit gibt Tab. 2.

**Tab. 2:** Überblick über Integrated Youth Mental Health Services (IYMHS) und ihre Strukturmerkmale. (Adaptiert nach [15, 25, 30]).

Programm	Land	Anzahl der Zentren (+ im Aufbau)	Altersgruppe	Anzahl der Nutzenden (Zeitraum)	Peer-Beteiligung	Job- und Schulcoaching	Behandlung somatischer Erkrankungen	Angebote für Angehörige	Spezialisierte Behandlung von Ersterkrankungen	Verlaufsevaluation mit klinischen Outcomes
Youth One-Stop Shops (YOSS)	Neuseeland	14	10–25	11.430 (MW pro Jahr)	+	+	+	+	–	+
Maison des Adolescents	Frankreich	104	11–25	700–1000 pro Zentrum pro Jahr	+	+	+	+	k. A.	+
Headspace	Australien	164 (+9)	12–25	106.574 (2020–2021)	+	+	+	+	+	+
Jigsaw	Irland	14 (+1)	12–25	>44.000 (seit 2008)	+	+	+	+		+
Community Health Assessment Team (CHAT)	Singapur	1	16–30	3343 (2009–2019)	+	–	–	k. A.	k. A.	k. A.
Headspace [5]	Dänemark	28 (+4)	12–25	k. A.	+	k. A.	+	+	–	Derzeit evaluiert
ACCESS Open Minds	Kanada	14	11–25	8043 Überweisungen und 5280 untersuchte Jugendliche (2016–2020)	+	+	–	+	k. A.	+
Headspace [16]	Israel	2	12–25	652 (1 Zentrum)	+	+	–	+	+	–
Foundry	Kanada	17 (+18)	12–24	4783 (2015–2018)	+	+	+	+	+	+
Mindlink [19]	Südkorea	16	15–30	206 (2019)	+	k. A.	k. A.	+	+	–
Youth Wellness Hubs Ontario	Kanada	25 (+5)	12–25	9585 (2020–2023)	+	k. A.	+	+	+	+
@ease [6, 22]	Niederlande	12 (+4)	12–25	>750 persönlich, > 3000 Online-Chats	+	k. A.	–	–	–	+, derzeit evaluiert
Soulspace [3, 4]	Deutschland	1	15–35	899 *transit* (2018–2020) 558 *FIT* (2016–2021)	+	+	–	+	+	–
Headspace*	Island	2 (+22 Satellitendienste)	12–25	390	k. A.	k. A.	k. A.	k. A.	k. A.	k. A.
Levelmind mit Headwind (Online-Service; [17])	Hongkong	8	12–25	>18.000	+	k. A.	k. A	k. A.	+	+
Piki [12]	Neuseeland	k. A.	18–25	5307 (2019–2020)	+	–	–	–	–	–
SODA [34]	Japan	1	12–35	105 (März bis September 2020)	k. A.	k. A.	–	+	k. A.	+
Allcove [33]	USA	2(+9)	12–25	5000 (2021–2023)	+	+	+	+	k. A.	k. A.

Originalquellen wurden nur dann zitiert, wenn in [15, 25, 30] keine Angaben dazu gemacht wurden

*k.* *A.* keine Angabe, *MW* Mittelwert

*Unveröffentlichte Daten

Das umfangreichste Beispiel für IYMHS ist das australische *headspace*-Programm mit aktuell 169 Zentren landesweit, gefolgt von Kanada mit verschiedenen Angeboten wie ACCESS Open Minds, Aire ouverte, Foundry oder Youth Wellness Hubs Ontario, Irland mit Jigsaw und den Niederlanden mit @ease (Tab. 2). Die meisten IYMHS sind in Ländern mit hohem Einkommen wie Australien, Kanada oder in Nordeuropa entstanden.

Die Angebote orientieren sich weitgehend an den in Tab. 1 genannten Prinzipien, sind niederschwellig, jugendfreundlich gestaltet, außerhalb von Krankenhäusern lokalisiert und adressieren Jugendliche und junge Erwachsene. Der Umsetzungsgrad und die Schwerpunktsetzungen unterscheiden sich jedoch teilweise [25]. Die meisten Angebote sind transdiagnostisch konzipiert und unterstützen auch bei sozialen oder beruflichen Problemlagen mit Sozialarbeit, Job- oder Schul‑/Ausbildungs-Coaching („supported employment and education“ [SEE]), einzelne adressieren auch körperliche Gesundheit [15, 30]. Einige Programme bestehen in erster Linie aus Angeboten von geschulten Peers oder Ehrenamtlichen (beispielsweise [22]), andere primär aus Angeboten von Fachkräften oder ausschließlich multiprofessionellen Teams (unter anderem [3]). Das Spektrum der Interventionen reicht von minimaler unterstützender Begleitung und basaler psychosozialer Beratung bis hin zur spezifischen Behandlung häufiger und schwerer psychischer Erkrankungen (etwa lösungsorientierte Kurztherapie, kognitive Verhaltenstherapie, Krisenintervention, medikamentöse Behandlung) und Vorhaltung spezialisierter multiprofessioneller Behandlung. Darüber hinaus bieten einige IYMHS Online-Dienste in Form von Online-Chats, Gruppenchats oder Online-Therapieangeboten (z. B. [1, 8]). Auch die adressierten Altersgruppen unterscheiden sich geringfügig, adressiert werden beispielsweise 12- bis 25-Jährige oder 15- bis 30-Jährige.

### Inanspruchnahme der Integrated Youth Mental Health Services

Laut ersten Daten werden die Angebote häufiger von Frauen als von Männern [22, 24, 26] und häufiger von älteren jungen Erwachsenen in Anspruch genommen [15]. IYMHS werden verhältnismäßig oft von Personen genutzt, die als „unterversorgt“ bzw. vulnerabel identifiziert werden (Personen mit Migrationshintergrund, aus einkommensschwachen Familien, ohne Erwerbstätigkeit oder nicht in Ausbildung, „lesbian, gay, bisexual, transgender, queer plus“ [LGBTQ+]; [15, 25]). Insgesamt werden die Zentren laut ihren Routinedaten von einer großen Anzahl der jungen Menschen aufgesucht (vergleiche Tab. 2).

Die von Nutzenden genannten Gründe für die Inanspruchnahme – mit Fokus auf psychische Gesundheit – werden in den Zentren unterschiedlich erhoben und umfassen unter anderem Ärger, Stress, Angstzustände, depressive Beschwerden, Stimmungsschwankungen, Substanzkonsum, Beziehungs- und Anpassungsprobleme, sexuelle Übergriffe und häusliche Gewalt sowie Probleme in der Schule einschließlich Mobbing. Die Zentren werden von einer erheblichen Anzahl junger Menschen mit Risikosymptomen für schwere psychische Erkrankungen, aber auch mit ersten psychotischen Episoden [21] oder mit Suizidalität aufgesucht [7, 13, 21].

### Evidenz zur Wirksamkeit der Integrated Youth Mental Health Services

Bisher liegen nur unkontrollierte Verlaufsstudien (Prä-post-Vergleiche) vor ([15, 25, 30]; Tab. 2). Eine erste quasiexperimentelle Studie mit „propensity score matching“ wird derzeit in Dänemark durchgeführt [5]. Junge Menschen berichten, dass sie von diesen Angeboten profitierten und sehr zufrieden mit der Beratung und/oder Behandlung waren [15, 25]. Für Personen mit schwereren Symptomen und für Personen, die weniger Leistungen erhalten haben, zeigen sich geringere Effekte. Sie haben weniger profitiert, was auf die Notwendigkeit einer Integration von bzw. Überweisung in spezialisierte Versorgung hinweist [15]. Aktuelle Evaluationsstudien aus den Niederlanden und Australien belegen eine klinisch relevante Verbesserung („reliable clinical change“) des psychosozialen Stresslevels, der sozialen und beruflichen Funktionalität sowie der Lebensqualität – je nach Outcome bei 9–40 % aller Nutzerinnen und Nutzer [6, 28].

### Aktuelle Situation in Deutschland

Die ersten IYMHS in Deutschland sind *soulspace* in Berlin [3, 4] und das in Aufbau befindliche *ancora* in Frankfurt [36]. In beiden IYMHS wird die niederschwellige, gemeindeintegrierte Angebotskomponente durch eine Finanzierung aus Landes- (Sozialgesetzbuch [SGB] IX, *soulspace*) oder Stiftungsmitteln (Crespo Foundation, *ancora*) zusätzlich zu den aus Krankenkassenmitteln (SGB V) finanzierten klinischen Leistungen der Institutsambulanzen der Erwachsenen- sowie der Kinder- und Jugendpsychiatrie erreicht.

*Soulspace* liegt in Berlin-Kreuzberg und ist ein Zusammenschluss von *transit* – Kontakt- und Beratungsstelle für junge Menschen der ajb GmbH Berlin und Teilen der Institutsambulanzen der Klinik für Psychiatrie, Psychotherapie und Psychosomatik des Vivantes Klinikums Am Urban (Erwachsenenpsychiatrie) und der Klinik für Kinder- und Jugendpsychiatrie, Psychotherapie und Psychosomatik des Vivantes Klinikums im Friedrichshain (Kinder- und Jugendpsychiatrie) unter einem Dach [4]. Die Beratungsstelle *transit* dient als erste Anlaufstelle, in der ohne Einlesen einer Krankenkassenkarte Einzel‑, Paar- und Familiengespräche sowie Kurse zu Stress- und Konfliktbewältigung angeboten werden. Bei Verdacht auf eine psychische Störung erfolgt eine Überleitung in den Ambulanzteil von *soulspace* zur spezifischen (Früh‑)Diagnostik und multiprofessionellen Behandlung oder eine Weitervermittlung in das weitere überwiegend ambulante Versorgungsnetzwerk. Das Ambulanzteam von *soulspace* arbeitet im Falle der Erstmanifestation einer schweren psychischen Störung eng und verzahnt mit dem spezialisierten Home Treatment zusammen, dem teilstationären und stationären Angebot des Frühinterventions- und Therapiezentrums FRITZ am Urban [31]. In *soulspace* sind die meisten der oben beschriebenen Strukturmerkmale für IYMHS erfüllt (Tab. 2). Erste Evaluationen deuten darauf hin, dass in soulspace wesentlich mehr junge Menschen der Erstkontakt mit dem Hilfesystem gelungen ist als in konventionellen Früherkennungsangeboten psychiatrischer Krankenhäuser [3].

## Diskussion

IYMHS werden von namenhaften Expertinnen und Experten gefordert, um jungen Menschen mit ersten psychischen Symptomen leichter zugängliche und akzeptablere Versorgungsangebote zu machen und damit die Chance zu erhöhen, die langfristigen Verläufe psychischer Erkrankungen zu verbessern. Erste Evaluationen zeigen, dass IYMHS von jungen Menschen erheblich in Anspruch genommen werden, diese jungen Menschen überwiegend zufrieden mit dem Angebot sind und sie häufig über relevante symptomatische, funktionelle und weitere subjektive Verbesserungen berichten [6, 15, 25, 28].

Kontrollierte Studien zur Wirksamkeit von IYMHS liegen noch nicht vor bzw. sind noch nicht abgeschlossen [5]. Dies ist zum Teil ethischen Überlegungen geschuldet, vor deren Hintergrund Forschende es als nicht akzeptabel bewerteten, erstmals hilfesuchende junge Menschen in eine Wartegruppe oder eine Gruppe ohne Behandlungsangebot zu randomisieren [7]. Auch ist der Zusammenhang zwischen den empfohlenen Strukturmerkmalen und Inanspruchnahme, klinischer Effektivität oder Zufriedenheit der Nutzenden bisher nicht empirisch untersucht. Diese Forschungslücke soll durch das von der Europäischen Union (EU) geförderte Projekt YOUTHreach, das Anfang 2025 gestartet ist, geschlossen werden. Das Ziel des Projekts besteht darin, einerseits die Wirksamkeit von IYMHS sowie zweier digitaler Mental-Health-Interventionen in sieben europäischen Ländern und Australien zu evaluieren [9]. Im Rahmen des YOUTHreach-Projekts wird auch soulspace in Berlin evaluiert, um die Evidenzlage für Deutschland zu erweitern. Dabei sollen die Strukturmerkmale verschiedener europäischer IYMHS in Bezug auf ihre Wirksamkeit quantitativ und qualitativ untersucht werden. Berücksichtigt werden dabei die Symptomatik (beispielsweise psychosozialer Stresslevel, Depression, Angst) und die soziodemografischen Merkmale der Nutzenden, um weitere Aufschlüsse über die Wirksamkeit bei bestimmten Personengruppen zu gewinnen.

Obwohl auch für Deutschland eine Reform der Versorgung hin zu mehr präventiven Angeboten für Jugendliche und junge Erwachsene gefordert wird [11, 29], existiert bisher mit *soulspace* in Berlin nur ein einziges aktives Zentrum und mit *ancora* in Frankfurt ein weiteres im Aufbau. In beiden Fällen wird die niederschwellige, gemeindeintegrierte Angebotskomponente durch eine Finanzierung aus Landesmitteln (*soulspace*) bzw. Stiftungsmitteln (*ancora*) zusätzlich zu den aus Krankenkassenmitteln finanzierten klinischen Angeboten der Institutsambulanzen der Erwachsenen- und der Kinder- und Jugendpsychiatrie erreicht. Eine weitere Möglichkeit zur Etablierung von IYMHS im deutschen Versorgungssystem liegt in der Zusammenführung von Krankenkassenmitteln und Mitteln des Öffentlichen Gesundheitsdiensts. Zwar sind in Deutschland in den letzten zwei Jahrzehnten vor allem in Großstädten verschiedene Früherkennungs- und Therapiezentren entstanden [2, 10]. Diese sind jedoch in der Regel an Krankenhäusern angesiedelt, auf die Identifizierung von klinischen Risikosymptomen einzelner Krankheitsbilder ausgerichtet und erreichen nur wenige Betroffene, die bislang außerhalb des Hilfesystems standen [23]. Die Früherkennungszentren könnten allerdings Ausgangspunkt für die Entwicklung weiterer IYMHS in Deutschland werden, wenn sie unter Beteiligung der Nutzenden um die Beratungskomponente erweitert und mit Unterstützung von Landes- oder Bundesmitteln an einem Standort außerhalb der Kliniken in der Kommune etabliert werden.

Zusammenfassend lässt sich feststellen, dass die Stärkung der Versorgung im Youth-Mental-Health-Bereich mit der Etablierung zahlreicher IYMHS international weit fortgeschritten ist, während Deutschland im internationalen Vergleich weit zurückliegt. Die Ergebnisse aus den ersten Pilotprojekten zeigen jedoch, dass IYMHS durch eine Zusammenführung von Krankenkassen- und Landes- oder Stiftungsmitteln, also durch die Realisierung einer vielfach geforderten sektorenübergreifenden Versorgung [20], auch im deutschen Versorgungssystem fruchtbar etabliert werden können. Auch wenn die Evaluationsdaten noch verbessert werden sollten, bietet der Aufbau von IYMHS die Hoffnung, jungen Menschen mit ersten psychischen Symptomen frühzeitig akzeptable Behandlungsangebote zu bieten, die den Krankheitsverlauf verbessern können und damit das Potenzial haben, viel Leid bei Betroffenen und ihren Angehörigen zu vermeiden und die erheblichen gesellschaftlichen Kosten zu reduzieren. Bei der Etablierung von IYMHS sollte die Versorgung von Personen mit ausgeprägterer Symptomatik differenzierter mitgedacht werden. Vor diesem Hintergrund sollten klinisches Personal und Gesundheitspolitik auf Krankenkassen‑, Landes- oder Bundesebene dringend ein Etablierungsprogramm für IYMHS in Deutschland erarbeiten.

## Fazit für die Praxis

- Während Länder wie Australien, die Niederlande, Dänemark und Irland bereits umfassende Strukturen der Integrated Youth Mental Health Services (IYMHS) etabliert haben, existieren in Deutschland bislang nur vereinzelte Pilotprojekte wie *soulspace* und *ancora*.
- *Soulspace* und *ancora* werden durch Landes- bzw. Stiftungsmittel zusätzlich zu Krankenkassenmitteln der Erwachsenen- sowie Kinder- und Jugendpsychiatrie finanziert und zeigen, dass sektorenübergreifende IYMHS-Modelle auch im deutschen Versorgungssystem realisierbar sind.
- Eine nationale Strategie zur flächendeckenden Implementierung der niederschwelligen Angebote fehlt hierzulande bislang.
- Bereits bestehende Früherkennungs- und Therapiezentren könnten als Ausgangspunkt für die Entwicklung weiterer IYMHS dienen. Durch die Erweiterung um Beratungsangebote und die Verlagerung in kommunale Strukturen könnten diese Zentren eine breitere Bedarfsgruppe erreichen und den Zugang für junge Menschen erleichtern.
- Verstärkte Forschung ist notwendig, um die Wirksamkeit und Nachhaltigkeit von IYMHS empirisch zu belegen und ihre Implementierung evidenzbasiert zu gestalten.

## References

[1] https://foundrybc.ca/virtual/. Zugegriffen: 2. Mai 2025

[2] https://www.psycho-check.com/zentren/deutschland/. Zugegriffen: 12. Apr. 2025

[3] Bechdolf A, Hanser S, Baumgardt J et al (2024) soulspace: Integrated youth mental health care in Berlin, Germany—An introduction to the program and a description of its users. Early Interv Psychiatry. 10.1111/eip.13522 38486399 10.1111/eip.13522

[4] Bechdolf A, Schellong M, Izat Y et al (2019) soulspace—Implementing a Low Threshold Specific Treatment and Early Intervention Programme for Young Adults and Adolescents in Routine Care in Germany. Psychiatr Prax 46:243–246. 10.1055/a-0918-9887 31269517 10.1055/a-0918-9887

[5] Bjørkedal STB, Christensen TN, Poulsen RM et al (2025) Study protocol: an effectiveness, cost-effectiveness, and process evaluation of headspace Denmark. Front Public Health. 10.3389/fpubh.2025.1491756 40260167 10.3389/fpubh.2025.1491756 PMC12009928

[6] Boonstra A, Van Amelsvoort T, Klaassen RMC et al (2024) Evaluating changes in functioning and psychological distress in visitors of the @ease youth mental health walk-in centres. BJPsych open 10:e101. 10.1192/bjo.2024.58 38699887 10.1192/bjo.2024.58 PMC11094437

[7] Boonstra A, Van Mastrigt G, Evers S et al (2023) ease peer-to-peer youth walk-in centres in The Netherlands: A protocol for evaluating longitudinal outcomes, follow-up results and cost-of-illness. Early Interv Psychiatry 17:929–938. 10.1111/eip.13443 37283500 10.1111/eip.13443

[8] https://headspace.org.au/eheadspace/. Zugegriffen: 2. Mai 2025

[9] https://youth-reach.eu/. Zugegriffen: 10. Apr. 2025

[10] https://www.dgppn.de/die-dgppn/referate/praevention-psychischer-erkrankungen.html. Zugegriffen: 16. Mai 2025

[11] DGPPN (2024) Versordung weitergedacht. Weiterentwicklung der psychiatrischpsychotherapeutischen Versorgung durch das Krankenhaus. https://www.dgppn.de/_Resources/Persistent/5e4a4c12fa547441b1be3adbad82b57dac17a9d9/20241107_DGPPN_Versorgungsmodell.pdf. Zugegriffen: 28. Apr. 2025

[12] Dowell A, Stubbe M, Gordon S et al (2021) Evaluation of the Piki Pilot Project (January 2020 – December 2021)—Final Report. In: Evaluation of the Piki Pilot Project (Integrated Therapies for 18–25 Year Olds): Final Report. University of Otago, Wellington

[13] Fusar-Poli P, Correll CU, Arango C et al (2021) Preventive psychiatry: a blueprint for improving the mental health of young people. World. Psychiatry, Bd. 20, S 200–221 10.1002/wps.20869 10.1002/wps.20869 PMC8129854 34002494

[14] GBD 2019 Mental Disorders C (2022) Global, regional, and national burden of 12 mental disorders in 204 countries and territories, 1990–2019: a systematic analysis for the Global Burden of Disease Study 2019. Lancet Psychiatry 9:137–150. 10.1016/s2215-0366(21)00395-3 35026139 10.1016/S2215-0366(21)00395-3 PMC8776563

[15] Hetrick SE, Bailey AP, Smith KE et al (2017) Integrated (one-stop shop) youth health care: best available evidence and future directions. Med J Aust 207(s18):5. 10.5694/mja17.00694 10.5694/mja17.00694 29129182

[16] Hoter-Ishay G, Mashiach-Eizenberg M, Roe D (2022) Young help-seeker profiles in Israel: The case of the first Israeli headspace centre. Early Intervention Psych 16:302–310. 10.1111/eip.13195 10.1111/eip.13195 34342140

[17] Hui CL, Suen YN, Lam BY et al (2022) LevelMind@JC: Development and evaluation of a community early intervention program for young people in Hong Kong. Early Interv Psychiatry 16:920–925. 10.1111/eip.13261 34894378 10.1111/eip.13261

[18] Killackey E, Hodges C, Browne V et al (2020) A Global Framework for Youth Mental Health: Investing in Future Mental Capital for Individuals, Communities and Economies. World Economic Forum, Geneva

[19] Kim SW, Kim JK, Jhon M et al (2021) Mindlink: A stigma-free youth-friendly community-based early-intervention centre in Korea. Early Interv Psychiatry 15:1389–1394. 10.1111/eip.13076 33233027 10.1111/eip.13076 PMC8451763

[20] Krüger U, Holke J, Brieger P et al (2022) Dialog zur Weiterentwicklung der Hilfen für psychisch erkrankte Menschen – auf dem Weg zur personenzentrierten Versorgung von psychisch erkrankten Menschen. https://www.bundesgesundheitsministerium.de/fileadmin/Dateien/5_Publikationen/Gesundheit/Berichte/Projektbericht_Dialog_gesamt_bf.pdf. Zugegriffen: 9. Mai 2025

[21] Leichsenring F, Steinert C, Rabung S et al (2022) The efficacy of psychotherapies and pharmacotherapies for mental disorders in adults: an umbrella review and meta-analytic evaluation of recent meta-analyses. – 21:133–145. 10.1002/wps.20941 10.1002/wps.20941 PMC8751557 35015359

[22] Leijdesdorff SMJ, Rosema S, Klaassen RMC et al (2022) Who is @ease? Visitors’ characteristics and working method of professionally supported peer-to-peer youth walk-in centres, anonymous and free of charge. Early Interv Psychiatry 16:1391–1397. 10.1111/eip.13294 35343056 10.1111/eip.13294 PMC10078703

[23] Leopold K, Nikolaides A, Bauer M et al (2014) Angebote zur Früherkennung von Psychosen und bipolaren Störungen in Deutschland. Nervenarzt 86:352–358. 10.1007/s00115-014-4119-2 10.1007/s00115-014-4119-2 25022895

[24] Mathias S, Tee K, Helfrich W et al (2022) Foundry: Early learnings from the implementation of an integrated youth service network. Early Intervention Psych 16:410–418. 10.1111/eip.13181 10.1111/eip.13181 PMC9292689 34008340

[25] Mcgorry PD, Mei C, Dalal N et al (2024) The Lancet Psychiatry. Comm Youth Ment Heal Lancet Psychiatry 11:731–774. 10.1016/s2215-0366(24)00163-9 10.1016/S2215-0366(24)00163-9 39147461

[26] O’reilly A, O’brien G, Moore J et al (2022) Evolution of Jigsaw—a National Youth Mental Health Service. Early Interv Psychiatry 16:561–567. 10.1111/eip.13218 34464507 10.1111/eip.13218

[27] Radez J, Reardon T, Creswell C et al (2021) Why do children and adolescents (not) seek and access professional help for their mental health problems? A systematic review of quantitative and qualitative studies. Eur Child Adolesc Psychiatry 30:183–211. 10.1007/s00787-019-01469-4 31965309 10.1007/s00787-019-01469-4 PMC7932953

[28] Rickwood D, Mceachran J, Saw A et al (2023) Sixteen years of innovation in youth mental healthcare: Outcomes for young people attending Australia’s headspace centre services. PLoS ONE 18:e282040. 10.1371/journal.pone.0282040 37390108 10.1371/journal.pone.0282040 PMC10313045

[29] Romanos M, Berg G, Brauer A et al (2024) How can we ensure the future care for children and adolescents with mental disorders? Bundesgesundheitsblatt Gesundheitsforschung Gesundheitsschutz 67:482–489. 10.1007/s00103-024-03858-w 38502362 10.1007/s00103-024-03858-w PMC10994988

[30] Settipani CA, Hawke LD, Cleverley K et al (2019) Key attributes of integrated community-based youth service hubs for mental health: a scoping review. Int J Ment Health Syst 13:52. 10.1186/s13033-019-0306-7 31367230 10.1186/s13033-019-0306-7 PMC6651922

[31] Siebert S, Leopold K, Baumgardt J et al (2022) Specialized inpatient treatment for young people with early psychosis: acute-treatment and 12-month results. Eur Arch Psychiatry Clin Neurosci 272:1–14. 10.1007/s00406-022-01379-8 35141809 10.1007/s00406-022-01379-8 PMC9508217

[32] Solmi M, Radua J, Olivola M et al (2022) Age at onset of mental disorders worldwide: large-scale meta-analysis of 192 epidemiological studies. Mol Psychiatry 27:281–295. 10.1038/s41380-021-01161-7 34079068 10.1038/s41380-021-01161-7 PMC8960395

[33] https://med.stanford.edu/psychiatry/special-initiatives/allcove.html. Zugegriffen: 5. Mai 2025

[34] Uchino T, Kotsuji Y, Kitano T et al (2022) An integrated youth mental health service in a densely populated metropolitan area in Japan: Clinical case management bridges the gap between mental health and illness services. Early Interv Psychiatry 16:568–575. 10.1111/eip.13229 34743415 10.1111/eip.13229

[35] Uhlhaas PJ, Davey CG, Mehta UM et al (2023) Towards a youth mental health paradigm: a perspective and roadmap. Mol Psychiatry 28:3171–3181. 10.1038/s41380-023-02202-z 37580524 10.1038/s41380-023-02202-z PMC10618105

[36] https://www.ancora-frankfurt.de/. Zugegriffen: 10. Apr. 2025

